# Preoperative and postoperative blood testosterone levels in patients with acromegaly: a prospective study

**DOI:** 10.3389/fendo.2023.1259529

**Published:** 2023-10-11

**Authors:** Duoxing Zhang, Xiaopeng Guo, Ming Feng, Xinjie Bao, Kan Deng, Yong Yao, Wei Lian, Bing Xing, Hanbi Wang

**Affiliations:** ^1^ Department of Neurosurgery, Peking Union Medical College Hospital, Chinese Academy of Medical Sciences and Peking Union Medical College, Beijing, China; ^2^ Peking Union Medical College, Chinese Academy of Medical Sciences and Peking Union Medical College, Beijing, China; ^3^ Key Laboratory of Endocrinology, China Pituitary Adenoma Specialist Council, Beijing, China; ^4^ Department of Obstetrics and Gynecology, Peking Union Medical College Hospital, Beijing, China

**Keywords:** acromegaly, male, low blood testosterone level, GH, surgery intervention

## Abstract

**Purpose:**

To investigate the prevalence of low blood testosterone level (LTL) and its determinant factors among active male acromegaly patients, as well as the effect of surgery on LTL in male acromegaly patients.

**Methods:**

A retrospective, single-center study focused on 252 male acromegaly patients aged 18 years–60 years diagnosed in the Peking Union Medical College Hospital from January 2015 to December 2018 was carried out. The measurements of preoperative and postoperative testosterone levels, serum growth hormone (GH), insulin-like growth factor 1 (IGF-1), and other clinical data were analyzed.

**Results:**

Forty per cent of subjects included were diagnosed with LTL pre surgery. Patients were divided into normal testosterone level (NTL) and LTL groups based on their testosterone level. There were significant differences (*p* < 0.01) between groups in the presence of macroadenomas, invasion of the cavernous sinus, compression of the optic chiasm, and serum GH and prolactin levels pre surgery. Invasion of the cavernous sinus [odds ratio (OR) = 4.299; *p* = 0.000] and serum prolactin level (OR = 1.023, *p* = 0.001) were independent predictors of LTLs in male patients before surgical intervention. A total of 67.9% of LTL patients recovered during the follow-up, with a new-onset rate of 3.4%. Body mass index, invasion of the cavernous sinus, GH, IGF-1, and prolactin levels, the presence of a prolactin-secreting tumor, and recovery from acromegaly were significantly different (*p* < 0.05) in the NTL group and in the LTL group during the follow-up. The presence of a prolactin-secreting tumor (OR = 0.224; *p* = 0.001) and recovery from acromegaly (OR = 0.168; *p* = 0.006) were independent predictors of LTLs in male acromegaly patients during the follow-up.

**Conclusion:**

The invasiveness of tumor and levels of blood prolactin are independent factors for LTLs before surgery, whereas GH and IGF-1 levels are not. Most male patients can recover from LTL after tumor restriction surgery: those who recover from acromegaly have a better chance of recovering from LTL.

## 1 Introduction

Acromegaly is a rare systemic illness (incidence of 5.3 per million person years and prevalence of 83 cases per million inhabitants) characterized by chronic hypersecretion of growth hormone (GH), in most cases caused by a GH secreting pituitary adenoma. GH hypersecretion leads to the overproduction of insulin-like growth factor 1 (IGF-1), which results in multisystem dysfunction such as somatic overgrowth, multiple comorbidities, physical disfigurement, and an increase in the rates of mortality (1, 2). One type of dysfunction commonly present in patients with acromegaly is hypogonadotropic hypogonadism (HH); it occurs in 30%–50% of acromegaly patients (3, 4). The influence of the pituitary adenoma on the secretion of follicle-stimulating hormone (FSH) and luteinizing hormone (LH) is one contributor to the insufficient gonadal function found in male and female acromegaly patients (5). Female patients diagnosed with HH may have menstrual cycle abnormalities, infertility, or decreased libido (6). Impaired testicular function, abnormal sperm production, and low blood testosterone level (LTL) can be found in male acromegaly patients diagnosed with HH, which is responsible for male infertility, an increase in body mass index (BMI), diminished physical strength, decreased libido, erectile dysfunction (ED), and loss of morning erection in these patients (7). Blood testosterone level plays an important part in the evaluation of HH in male patients (8), as LTL is a direct sign of, and a factor responsible, for HH (9).

The effects of LTL take many forms, which include loss of muscle mass, central obesity, and reduced bone density in aging male patients (10). Testosterone replacement therapy (TRT) has been considered a first-line therapy associated with LTL in aged males (11). Monteiro et al. evaluated the relationship between HH and non-function pituitary adenoma (NFPA) pre and post surgery (12), yet few studies have evaluated LTL in acromegaly patients both before and after pituitary adenoma restriction surgery.

The aim of this study was to investigate the prevalence and independent determinant factors of LTL among male acromegaly patients and the effect of surgery on LTL during follow-up.

## 2 Subjects and methods

### 2.1 Study cases

A total of 252 men aged 18 years–60 years who had been diagnosed with pituitary adenoma that caused acromegaly and who received neurosurgery at the Peking Union Medical College Hospital (PUMCH) from January 2015 to December 2018 were included in this retrospective, single-center study. The diagnosis of acromegaly was based on the Endocrine Society’s clinical practice guidelines (13), and LTL was defined as a serum total blood testosterone level below 1.75 ng/mL after measurement on two consecutive mornings (14). This study was carried out in accordance with the tenets of the Helsinki declaration and was approved by the Institutional Review Board at PUMCH. All patients provided informed consent before enrollment.

Demographic information (age at diagnosis, sex, disease duration, and BMI), tumor magnetic resonance imaging (MRI) features (diameter, cavernous sinus invasion, sphenoid sinus invasion, and optic chiasm compression), and endocrine results [fasting GH, GH nadir after the oral glucose tolerance test (OGTT), fasting IGF-1, prolactin, total blood testosterone levels] were recorded. Disease duration was defined as the interval from the onset of acromegalic presentations to clinical diagnosis. Macroadenoma was defined as a tumor with a diameter ≥ 10 mm. On the coronal plane, a Knosp grade (15) of 3 or 4 on either side indicated tumor invasion into the cavernous sinus.

### 2.2 Surgery for pituitary adenomas and patient follow-up

All enrolled patients received pituitary tumor resection surgery: tumors were pathologically inspected by histopathological and immunohistochemical (IHC) staining. Routine postoperative follow-up, which began 3 months after surgery, was scheduled for all patients. The follow-up evaluations included serum random GH level, IGF-1 level, nadir GH after OGTT, serum prolactin level, blood testosterone level, and pituitary MRI. The remission of acromegaly was considered to have been achieved if the random GH level was < 1 ng/mL or the GH nadir was < 0.4 ng/mL and the IGF-1 level was normal (16). The recovery from LTL was considered to have been achieved if the measured testosterone level was > 1.75 ng/mL on two consecutive mornings. For cured patients, we recommended a follow-up frequency of every 6 months or 1 year. For patients who had a recurrence, follow-up was ceased and a secondary surgery, somatostatin analog treatment, or gamma knife treatment was recommended based on the individual’s needs. The follow-up duration was defined as the interval from surgery to the last follow-up for cured patients or the time interval from surgery to recurrence for patients experiencing recurrence.

### 2.3 Statistical analyses

Statistical analyses were performed using SPSS, version 27.0 (IBM Corporation, Armonk, NY, USA). The results are expressed as percentages or mean ± standard deviation (SD). A Kolmogorov–Smirnov test was used to evaluate the normality of the distribution of each parameter. Comparisons of categorical variables and proportions were analyzed using a chi-squared test. An unpaired *t*-test was used to assess the differences between the two groups in variables with normally distributed values, and a Mann–Whitney *U*-test was used to examine differences between groups in variables lacking normal distributions. Binary logistic regression was used to analyze covariates between LTLs before and during follow-up with different characteristics. A *p*-value < 0.05 was considered statistically significant (17).

## 3 Results

### 3.1 General characteristics and clinical features of patients

Demographic, tumor-related, and endocrine-related information is presented in **Table 1**. A total of 252 male patients aged 18 years–60 years had a mean age of 38.5 years ± 10.35 years and a BMI of 27.5 kg/m^2^ ± 3.71 kg/m^2^. Disease duration (defined as the time since the first symptoms connected with acromegaly to a definitive diagnosis) ranged from 1 month (found by routine medical examinations) to 480 months (mean 82.9 months ± 74.22 months). Macroadenomas (81.7%, 206/252) were more often present than microadenomas in the study group. Overall, 26.6% (67/252) of tumors invaded the cavernous sinus, and 28.2% (71/252) compressed the optic chiasm. The nadir GH level was 39.2 ng/mL ± 98.05 ng/mL (mean ± SD; range 1.7 ng/mL–970.0 ng/mL) and the IGF-1 level was 959 ng/mL ± 273.85 ng/mL (range 194.0 ng/mL–1,556.0 ng/mL). In addition, 40% (101/252) of patients were diagnosed as having LTLs. Only 8.3% (21/252) of patients reported a decreased libido. Deficiencies in other pituitary target organ axes included a 6.7% (17/252) deficiency rate in the adrenocorticotropic hormone (ACTH)–cortisol axis and a 3.2% (8/252) deficiency rate in the thyroid-stimulating hormone (TSH)–thyroid axis. Osteoporosis was present in 2.4% (6/252) of patients and diabetes mellitus was found in 24.6% (62/252) of patients.

**Table 1:** Clinical and endocrine features of the 252 active male acromegaly patients.

	Values
Age at diagnosis (years)	38.5 ± 10.35
BMI (kg/m^2^)	27.5 ± 3.71
Disease duration, months	82.9 ± 74.22
Macroadenoma, *n* (%)	206 (81.7)
Cavernous sinus invasion, *n* (%)	67 (26.6)
Compression of the optic chiasm, *n* (%)	71 (28.2)
Microscopic transsphenoidal approach, *n* (%)	192 (76.2)
Endoscopic transsphenoidal approach, *n* (%)	55 (21.8)
Transcranial approach, *n* (%)	2 (0.8)
GH level (ng/mL)	39.2 ± 98.05
IGF-1 level (ng/mL)	959 ± 273.85
Prolactin+ on IHC staining, *n* (%)	95 (37.7)
Acromegaly remission, *n* (%)	161 (63.9)
Decreased libido, *n* (%)	21 (8.3)
ACTH–cortisol deficiency, *n* (%)	17 (6.7)
TSH–thyroid deficiency, *n* (%)	8 (3.2)
Osteoporosis, *n* (%)	6 (2.4)
Diabetes mellitus, *n* (%)	62 (24.6)
Overall preoperative testosterone deficiency, *n* (%)	101 (40)

Continuous variables are presented as means ± SDs if normally distributed or median (25th and 75th quartile) if not normally distributed. BMI, Body Mass Index; GH, random Growth Hormone; IGF-1, Insulin-like Growth Factor 1; Prolactin+ on IHC staining, prolactin secrecting tumor positive found on immunohistochemistry staining; ACTH, Adrenocorticotropic hormone; TSH, Thyroid Stimulating Hormone.

### 3.2 Clinicopathological correlations of normal-blood-testosterone-level and low-blood-testosterone-level patients pre surgery

A total of 252 male acromegaly patients with presurgical testosterone records were divided into two groups, normal testosterone levels (NTLs) (151/252) and LTL (101/252), as listed in **Table 2**. Comparisons between the NTL and LTL groups were performed, including comparing general features such as age at diagnosis, BMI, and disease duration. Image characteristics compared included the macroadenoma occurrence rate, cavernous sinus invasion, and compression of the optic chiasm. Other features such as having complications and decreased libido, and random GH levels, IGF-1 levels and prolactin levels were also compared. Among them, the percentages of macroadenomas (115/151, 76.2% *vs*. 91/101, 90.1%; *p* = 0.007), invasions of the cavernous sinus (23/151, 15.2% *vs.* 44/101, 43.6%; *p* = 0.000), and compressions of the optic chiasm (29/151, 19.2% *vs.* 42/101, 41.6%; *p* = 0.000) suggest a larger tumor size and greater invasion capacity, and show significant differences between the two groups. The levels of random GH (29.0 ng/mL ± 84.60 ng/mL *vs.* 54.0 ng/mL ± 114.08 ng/mL; *p* = 0.000) and prolactin levels before surgery (37.21 ng/mL ± 85.18 ng/mL *vs.* 13.44 ng/mL ± 13.29 ng/mL; *p =* 0.050) also show significant differences. No significant differences were found between the NTL and LTL groups for general factors such as age at diagnosis, BMI, and disease duration. A reported decrease of libido and having complications were not significantly different between the two groups.

**Table 2:** Clinicopathological correlations of NTL and LTL patients in acromegaly.

	NTL (n = 151)	LTL (n = 101)	*p*-value
Age at diagnosis (years)	38.5 ± 10.69	38.6 ± 9.87	0.966
BMI (kg/m^2^)	27.2 ± 3.71	28.0 ± 3.73	0.092
Disease duration (months)	80.9 ± 75.54	85.88 ± 72.48	0.439
Macroadenoma, *n* (%)	115 (76.2)	91 (90.1)	0.007
Cavernous sinus invasion, *n* (%)	23 (15.2)	44 (43.6)	0.000
Compression of the optic chiasm, *n* (%)	29 (19.2)	42 (41.6)	0.000
Complications, *n* (%)	77 (51.0)	54 (53.5)	0.353
Decreased libido, *n* (%)	10 (6.6)	11 (10.9)	0.230
GH level (ng/mL)	29.0 ± 84.60	54.0 ± 114.08	0.000
IGF-1 level (ng/mL)	941 ± 269.41	985 ± 279.65	0.150
Serum prolactin level (ng/mL)	37.21 ± 85.18	13.44 ± 13.29	0.050

Continuous variables are presented as means ± SDs if normally distributed or median (25th and 75th quartile) if not normally distributed.

### 3.3 Predictors of low blood total testosterone level before surgery

The patients in **Table 2** were further analyzed for individual risk factors of LTLs among acromegaly patients. The macroadenoma occurrence rate, cavernous sinus invasion, compression of the optic chiasm, and blood GH and prolactin levels were analyzed using a binary logistic regression. The results of the regression analyses are shown in **Table 3**. The invasion of the cavernous sinus (OR = 4.299; *p* = 0.000) and blood prolactin levels (OR = 1.023; *p* = 0.001) were considered important predictors of LTLs in acromegaly patients before surgical intervention. Other factors that represent the size of the tumor, including tumor size, compression of the optic chiasm, and GH level, however, were not shown to predict LTLs before surgery.

**Table 3:** Predictors of low blood total testosterone level before surgery.

	OR	95% CI	*p*-value
Macroadenoma, *n* (%)	2.770	1.302–5.891	0.611
Cavernous sinus invasion, *n* (%)	4.299	2.336–7.655	0.000
Compression of the optic chiasm, *n* (%)	2.946	1.671–5.191	0.234
GH level (ng/mL)	1.002	0.999–1.006	0.775
Serum prolactin level (ng/mL)	1.023	1.009–1.038	0.001

### 3.4 Effect of tumor resection on testosterone levels in acromegaly patients

**Figure 1:**
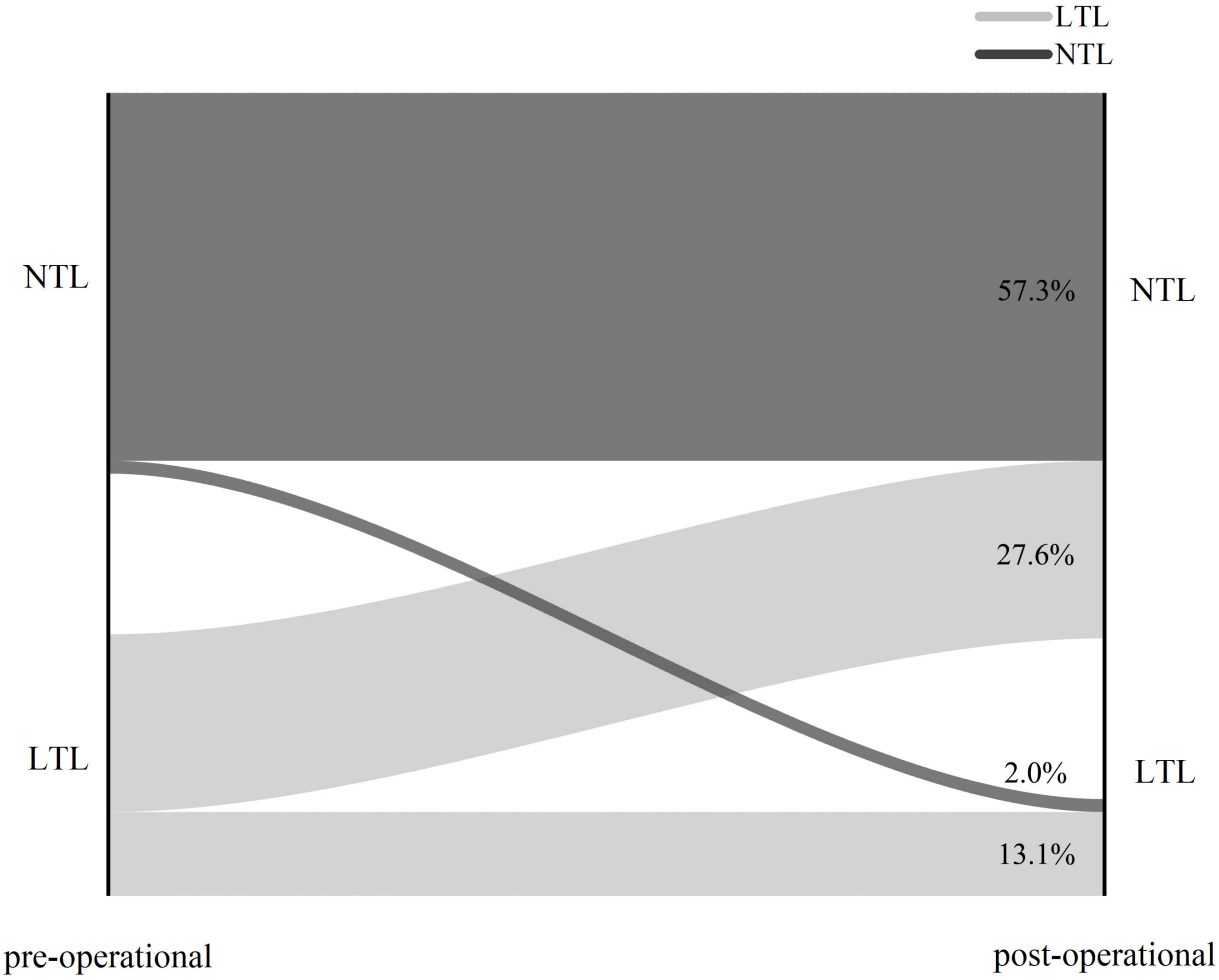
Sankey graph of surgery outcome of 199 acromegaly male patients. NTL: Normal Testosterone Level. LTL: Low Testosterone Level.

Median follow-up time after surgery was 45 months (3 months–80 months). Out of the 252 male acromegaly patients, 199 had both presurgical and long-term follow-up records of blood testosterone levels, we analyzed and classified them into NTL (Normal Testosterone Level) group and LTL (Low Testosterone Level) groups based on testosterone levels. 81 of 199 patients suffered LTL preoperatively while 118 of them had NTL preoperatively. Recovery status of all 199 patients were shown in **Figure 1**. A total of 55 out of 81 patients which suffers LTL preoperatively recovered and reached NTL post-operational (remission rate 67.9%, 27.6% (55/199)), whereas 26 of them (32.1% (26/81), 13.1% (26/199)) remained LTL postoperatively. 4 out of 118 NTL patients were found to have LTL during follow-up (new onset rate 3.4%, 2.0% (4/199)), whereas rest of NTL patients remained NTL postoperatively (57.3% (114/199)).

### 3.5 Clinicopathological correlations of NTL and LTL patients after surgery

Eighty-one LTL patients with both preoperative and follow-up serum testosterone records were divided into NTL (55/81) and LTL groups (26/81) after their operation (listed in **Table 4**). Factors including age at diagnosis; BMI; disease duration; MRI characteristics such as macroadenoma occurrence rate, cavernous sinus invasion, and compression of the optic chiasm; having complications; decreased libido; endocrine features GH; IGF-1 levels; serum prolactin levels; the pathology factor of prolactin secretin tumors; and recovery from acromegaly were compared. There were significant differences in terms of BMI (26.96 kg/m^2^ ± 2.71 kg/m^2^ *vs*. 30.0 kg/m^2^ ± 5.13 kg/m^2^; *p* = 0.001), invasion of the cavernous sinus (18/55, 32.7% *vs.* 16/26, 61.5%; *p* = 0.014), levels of GH at follow-up (2.03 ng/mL ± 2.47 ng/mL *vs.* 8.48 ng/mL ± 14.42 ng/mL; *p* = 0.013), IGF-1 levels at follow-up (389.7 ng/mL ± 211.26 ng/mL *vs.* 583 ng/mL ± 378.00 ng/mL; *p* = 0.042); prolactin levels at follow-up (7.65 ng/mL ± 4.66 ng/mL *vs.* 34.40 ng/mL ± 77.29 ng/mL; *p* = 0.021), occurrence of prolactin secretin tumors (19/55, 34.5% *vs.* 3/26, 11.5%; *p* = 0.023), and recovery from acromegaly (41/55, 74.5% *vs.* 9/26, 34.6%; *p* = 0.000) between the two groups during the follow-up. No significant differences were found between the NTL and LTL groups for general factors such as age at diagnoses, disease duration, and other size indication factors such as percentage of macroadenoma and compression of the optic chiasm. The endocrine factors before surgery were not significantly different between two groups.

**Table 4:** Clinicopathological and endocrinological correlations of NTL and LTL after surgery.

	NTL (*n* = 55)	LTL (*n* = 26)	*p*-value
Age at diagnosis (years)	39.44 ± 9.51	36.27 ± 9.97	0.207
BMI (kg/m^2^)	26.96 ± 2.71	30.07 ± 5.13	0.001
Disease duration (months)	80 ± 61.63	106 ± 93.15	0.281
Macroadenoma, *n* (%)	51 (92.7)	24 (92.3)	0.946
Cavernous sinus invasion, *n* (%)	18 (32.7)	16 (61.5)	0.014
Compression of the optic chiasm, *n* (%)	19 (34.5)	14 (53.8)	0.099
Complications, *n* (%)	29 (52.7)	14 (53.8)	0.925
GH level—initial (ng/mL)	37.78 ± 35.59	72.43 ± 177.68	0.491
GH level—follow-up, ng/mL)	2.03 ± 2.47	8.48 ± 14.42	0.013
IGF-1 level—initial (ng/mL)	1013.6 ± 260.76	973.9 ± 323.19	0.754
IGF-1 level—follow-up (ng/mL)	389.7 ± 211.26	583 ± 378.00	0.042
Serum prolactin level—initial (ng/mL)	23.24 ± 25.81	53.04 ± 115.43	0.607
Serum prolactin level—follow-up (ng/mL)	7.65 ± 4.66	34.40 ± 77.29	0.021
Prolactin+ on IHC staining, *n* (%)	19 (34.5)	3 (11.5)	0.023
Remission of GH level, *n* (%)	41 (74.6)	9 (34.6)	0.000

Continuous variables are presented as means ± SDs if normally distributed or median (25th and 75th quartile) if not normally distributed.

### 3.6 Risk factors for low testosterone level before surgery intervention

The patients in **Table 4** were further analyzed for independent risk factors of LTL recovery among male acromegaly patients after surgery. Significant differences between the NTL and LTL groups in terms of BMI, invasion of the cavernous sinus, GH level at follow-up, IGF-1 level at follow-up, prolactin level at follow-up, occurrence of prolactin secretin tumors, and recovery of acromegaly are analyzed in **Table 5**. The presence of a prolactin secretin tumor (OR = 0.224; *p* = 0.001) and recovery of acromegaly (OR = 0.168; *p* = 0.006) were the only two independent predictors of LTL in acromegaly patients after surgical intervention. The size of the tumors and endocrine factors that implied the recovery of acromegaly were not independent factors that influenced testosterone level recovery after surgery.

**Table 5:** Determining factors of low serum testosterone recovery after surgery.

	OR	95% CI	*p*-value
BMI (kg/m^2^)	1.260	1.084–1.465	0.064
Cavernous sinus invasion, *n* (%)	0.304	0.115–0.802	0.679
GH level—follow-up (ng/mL)	1.152	1.010–1.315	0.192
IGF-1 level—follow-up (ng/mL)	1.002	1.001–1.004	0.973
Serum prolactin level—follow-up (ng/mL)	1.101	1.013–1.196	0.135
Prolactin+ on IHC staining, *n* (%)	0.224	0.058–0.867	0.001
Remission of GH, *n* (%)	0.168	0.060–0.466	0.006

## 4 Discussion

Acromegaly is a systemic disease associated with increased morbidity and patients presenting with cardiovascular, metabolic, respiratory, endocrine, articular, and bone complications. Most of these comorbidities could be prevented with treatments (18), and personalized designed methods that completely removed the tumor mass were the most helpful method among them (19).

In this study, the mean age at diagnosis was 38.5 years, which was similar with previous study (20–25), which indicates a mean age at diagnosis of 36.5 years–48.5 years. The median disease duration was 82.9 months, which is longer than in a previous study (54 months–60 months), but the wide range suggested that this could more be of a personal recall bias and depend on subjective patient measures. The mean BMI was 27.5 kg/m^2^, which is classified overweight (26). This may have been due to the lipolytic and insulin resistance effects of GH and IGF-1, which results in the mobilization of free fatty acids and the resistance of blood glucose found in acromegaly patients (27). Macroadenoma was present in most patients (81.7%), which was consistent with previous research (15, 17, 28). A long disease duration and delayed diagnoses of the disease may facilitate tumor growth. The MRI characteristics that implied cavernous sinus invasion (26.6%) and compression of the optic chiasm (28.2%) were found only in a minority of patients, which is similar to previous research (29). A majority of the patients received a microscopic transsphenoidal approach (76.2%) and an endoscopic transsphenoidal approach (21.8%), which were minimally invasive procedures performed by an experienced neurosurgeon; only 2 out of 252 patients (0.8%) received a transcranial approach due to the failure of resection tumor via a minimally invasive approach. The random GH levels (39.2 ng/mL ± 98.05 ng/mL) and IGF-1 levels (959 ng/mL ± 273.85 ng/mL) in active male acromegaly patients were higher than in the normal population. The pathology findings revealed that prolactinoma was found in 95 acromegaly patients (37.7%). The acromegaly remission rate reached 63.9%, which was consistent with the results of an investigation by Araujo-Castro et al. (30), for which the overall rate of surgical remission with the 2000 criteria was 73.5% and 51.0% with the 2010 criteria. Decreased libido was experienced by 21 patients (8.3%) and the overall preoperative testosterone deficiency levels were experienced by 101 patients (40%).

The percentages of macroadenomas, invasion of the cavernous sinus, and compression of the optic chiasm in the LTL group were significantly higher than in NTL group. This suggests that male acromegaly patients with a larger and more invasive tumor would also have a greater chance of LTL, which can be explained by the size effect of tumor on the normal function of the pituitary gland by changing pituitary architecture and changing blood flow (31). Endocrine factors, including blood GH and prolactin levels before surgery, were also significantly different between the NTL and LTL groups. Rie Nishio et al. (32) reported that excessive GH was the most relevant factor for hypogonadism in male acromegaly patients, whereas blood total testosterone level and free testosterone level had the strongest correlation with GH in acromegaly patients. Hyperprolactinemia was likely to be caused by the co-secretion of GH and prolactin from the tumor (33). Although older age and obesity had been considered to be important determining factors of LTL in previous studies (34, 35), no significant differences were found between the NTL and LTL groups for general factors such as age at diagnosis, BMI, and disease duration. This is likely to be due to the fact that previous studies had been conducted in non-acromegalic patients, and unlikely to have taken into account the effects of GH and IGF-1 on overall health. The reported decrease of libido and having complications were not significantly different between the two groups.

The invasion of the cavernous sinus (OR = 4.299; *p* = 0.000) and serum prolactin levels (OR = 1.023; *p* = 0.001) were considered independent predictors of LTLs in male acromegaly patients before surgical intervention. Hyperprolactinemia may cause male hypogonadism (36). It was generally believed that the mechanism of hyperprolactinemia-induced sexual dysfunction was caused by a decrease in testosterone secretion (37), whereas the mechanism could partly be explained by the influence of prolactin on serotonin systems in the brain.

A total of 55 out of 81 (67.9%) LTL patients recovered after surgery, but 26 out of 81 (32.1%) retained LTLs. Of the 118 NTL patients before surgery, four (3.4%) developed a new-onset LTL during follow-up, whereas the rest of the patients retained NTLs. In a previous retrospective study of endocrine function after transsphenoidal surgery for non-functional pituitary adenomas (38), laboratory normalization rates of the male reproductive axis 6 weeks and 6 months after surgery without hormone replacement were 26% and 36%, respectively. In our study, the rate of recovery after 6 months was higher than that of the previous study of non-functional pituitary adenomas, suggesting that acromegaly patients recover better on their reproductive axis after the removal of a GH-releasing tumor. This can be explained by the effect of the GH and IGF-1 on the reproductive axis. The removal of the tumor not only releases normal pituitary tissue from compression of tumor tissue, but it also retains normal pituitary function by removing excess GH and IGF-1 in the subject’s blood that affects FSH/LH secretion. In a study by Behre et al. (39), intramuscular substitution therapy was applied to 52 patients, through a 250-mg testosterone enanthate injection almost every 3 weeks. In their study, TRT yielded a normalization of testosterone serum levels in all patients for up to 16 years. In a meta-analysis by Varanoske et al. (40), 24 articles were analyzed and it was found that testosterone administration significantly increased serum testosterone levels in all individuals. In our study, patients who were treated surgically did not receive additional testosterone, and the remission rate for LTLs after surgery was 67.9% in the long term without additional testosterone administration. This suggests that testosterone was needed for those patients who were initially diagnosed as having LTL and were further classified as having primary or secondary hypogonadism; treatment for them might be different accordingly (14). For patients with LTL, if both LH and FSH levels were elevated, they could be classified as having primary hypogonadism; for those with unelevated LH and FSH levels, they could be classified as having secondary hypogonadism, which is closely associated with obesity and the hypothalamus-pituitary axis (41). The results from our study suggest that for the secondary hypogonadism patients with known cause of LTLs, the restriction of the tumor can be an effective way of restoring testosterone to a normal range.

There were significant differences in the BMI, invasion of the cavernous sinus, GH, IGF-1, and prolactin levels at follow-up, occurrence of prolactin-secreting tumors, and recovery from acromegaly between the NTL and LTL groups after surgery. Men with obesity alone tend to have LTLs (42). This could explain why the LTL group tended to have a higher average BMI. We found that the LTL group after surgery had a higher percentage of invasion tumors, suggesting that the restriction of the tumor might not be thorough; in these cases tumor mass left unrestricted might cause LTLs. GH, IGF-1, and prolactin inhibit the production of LH and FSH and the function of testicles, as mentioned before; this could explain the difference between the two groups. Prolactin and prolactinoma, which produce prolactin, can cause hypogonadism (43). The higher serum prolactin level found in LTL patients was related to higher prolactin levels. More patients in the NTL group had prolactin (+) cells after IHC staining; this may be explained by the fact that surgery can remove most of the prolactin-secreting component in the tumor mass so the cells could be found in the staining. Recovery from acromegaly had a direct effect on recovery of testosterone levels for suppression of testosterone production due to the effect of excess GH and IGF-1 was removed.

The occurrence of a prolactin-secreting tumor (OR = 0.224; *p* = 0.001) and recovery from acromegaly (OR = 0.168; *p* = 0.006) were considered to be independent predictors of LTL after surgery. It was hard to understand that only the level of prolactin in blood could not be a predicting factor, whereas detection of a prolactin-secreting tumor on IHC staining might reduce the risk of LTLs. Because excess prolactin is correlated with tumor size (44), the portion and absolute size of prolactinoma in a GH adenoma, which is correlated with the level of prolactin in blood, should also be correlated with the prognosis of testosterone level.

Sex hormone-binding globulin was not evaluated in this research, which can be considered a limitation of this study. Further study with regard to other hormones, such as FSH and LH, and standards including erection time and semen quality is required. Due to the limited sample size, improvement can be made in the statistics after surgery regarding follow-up rates and testosterone levels.

## 5 Conclusions

Invasiveness of the tumor and the level of blood prolactin are independent factors for LTLs before surgery, whereas the GH and IGF-1 levels are not. Most male patients can recover from LTLs after tumor restriction surgery, and those who recover from acromegaly have a better chance of recovery from LTL.

## Data availability statement

The raw data supporting the conclusions of this article will be made available by the authors, without undue reservation.

## Author contributions

DZ: Conceptualization, Data curation, Formal Analysis, Investigation, Methodology, Project administration, Software, Supervision, Validation, Writing – original draft, Writing – review & editing. XG: Writing – review & editing. MF: Writing – review & editing. XB: Writing – review & editing. KD: Writing – review & editing. YY: Writing – review & editing. WL: Writing – review & editing. BX: Writing – review & editing. HW: Writing – review & editing.
